# A smartphone application to objectively monitor music listening habits in adolescents: Personal listening device usage and the accuracy of self-reported listening habits

**DOI:** 10.1186/s40463-020-00488-5

**Published:** 2021-02-15

**Authors:** Danique E. Paping, Jantien L. Vroegop, Simone P. C. Koenraads, Carlijn M.P. le Clercq, André Goedegebure, Robert J. Baatenburg de Jong, Marc P. van der Schroeff

**Affiliations:** 1 Department of Otorhinolaryngology, Head and Neck Surgery, Erasmus University Medical Center, Rotterdam, the Netherlands; 2 The Generation R Study Group, Erasmus University Medical Center, Rotterdam, the Netherlands

**Keywords:** Personal listening device, Smartphone application, Noise-induced hearing loss, Music, Listening habits, Behaviour

## Abstract

**Background:**

Listening to music through personal listening devices (PLDs) has become more prevalent during last decades. The aim of this study was to evaluate music listening habits through PLDs in adolescents with a smartphone application, and to assess the accuracy of self-reported listening habits.

**Methods:**

This study was embedded in the Generation R Study, a population-based prospective birth cohort in Rotterdam, the Netherlands. A smartphone application for Android operating systems was developed to objectively monitor music listening habits for a period of 35 days. A postal questionnaire was used to subjectively assess listening habits. The level of agreement between the objectively measured and self-reported listening habits were evaluated using weighted kappa coefficients. Data were collected from May 2017 to March 2019.

**Results:**

A total of 311 adolescents aged 12 to 15 years were included, of whom 237 (76.2%) completed the postal questionnaire. The results of the smartphone application showed that the median listening frequency was 2.1 days a week (IQR 1.0–3.4), the median listening time 21.1 min a day (IQR 9.1–53.7), and the mean listening level 54.5% (SD 18.1%). There was a slight to fair agreement between the objectively measured, and self-reported listening habits according to the weighted kappa coefficients (k = 0.179 to 0.364).

**Conclusions:**

The results of the current study suggest that self-reported measures of listening habits are not always accurate. We consider a smartphone application to monitor listening habits of added value in future research investigating the possible damaging effects of PLDs on hearing acuity.

**Graphical abstract:**

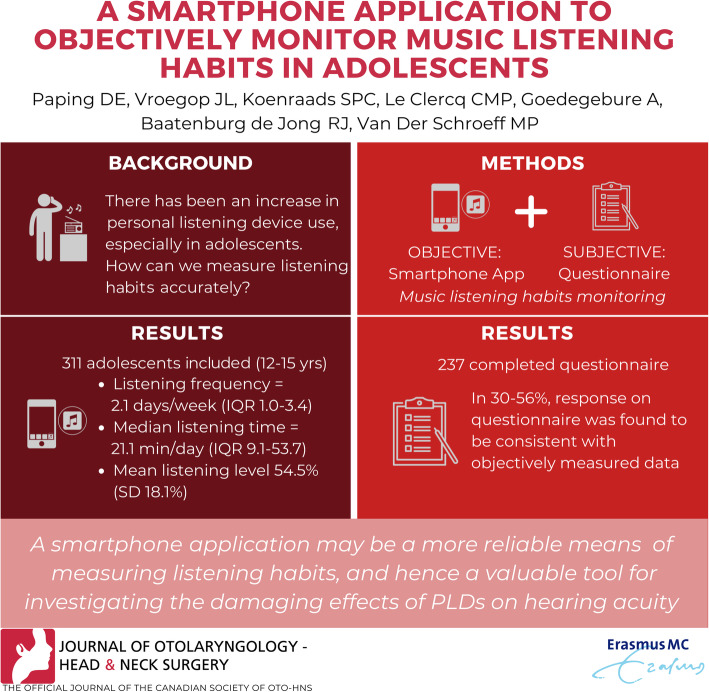

**Supplementary Information:**

The online version contains supplementary material available at 10.1186/s40463-020-00488-5.

## Background

Noise-induced hearing loss has long been recognized as an occupational disease. However, the incidence of hearing loss as a result of occupational noise exposure seems to be decreasing in the developed countries, due to preventive measures [1, 2]. Meanwhile, there is a growing concern about the potential risk leisure noise exposure poses on hearing, especially in young people [3]. Various sources of leisure noise exposure have become widespread among the general population, including listening to music with headphones, visiting concerts, festivals, and clubs [4–6]. Around 90% of adolescents and young adults use a personal listening device (PLD), with smartphones the most commonly used [7–11]. Music has played an important role in our society for decades, but the evolution of technology changed the way we listen. Music streaming services offer users millions of songs, and with good sound quality, music can be played at a high volume without distortion. Maximum volume levels of smartphones have been reported to range from 75 to 126 A-weighted decibels (dB(A)), depending on the type of smartphone, and style of headphones used [12–14]. Listening to music until 80 dB(A) for a maximum of 40 h a week may be regarded as safe [15]. As sound at a sufficient intensity and duration can cause damage to the inner ear, millions of people who are listening to music with their PLDs are potentially at risk of developing hearing problems [16, 17]. In order to assess this potential risk of noise-induced hearing loss due to PLDs, it is essential to evaluate the listening habits of PLD users in everyday life.

Over the last few years, a substantial amount of literature examined the listening habits of PLD users. These were evaluated by using self-reports, and physical measurement of preferred listening levels using an ear simulator or a miniature microphone inserted in the subject’s ear canal [5]. Studies using self-reports show that it common for people aged 12 to 25 years to listen multiple times a week, for more than 1 h a day, and at a medium to high listening level [7–10, 18–21]. However, the accuracy with which PLD users report on their listening habits is questionable. Self-reports are convenient and low-cost, but sensitive to misreporting and bias. In addition, single measurements of preferred listening levels do not take into account that listening levels change as a function of the background noise level and over time [22, 23]. Considering these drawbacks, the validity of questionnaires as a tool to measure noise exposure from PLDs remains to be determined.

Portnuff et al. was the first to compare actual measured listening habits to self-reported data [24]. Twenty-four participants aged 19 to 29 years had their listening habits monitored for a period of 1 week. An analog splitter was plugged into the output jack of the PLD, with one end connected to the earphones and the other end to an external dosimeter. The participants in the study reported their listening habits with reasonable accuracy. Moderate to strong correlations were found between the objective and self-reported measures. However, an important limitation of the methodology used by Portnuff et al. is that an external dosimeter had to be carried along with the PLD. Besides that it might be uncomfortable, it is a constant reminder of listening habits being monitored, thereby possibly influencing listening habits and the accuracy with which participants report on their listening habits. In the study of Kaplan-Neeman et al., a smartphone application was used to monitor listening habits of 37 young adults aged 18 to 32 years [25]. Participants received a smartphone, preinstalled with the application and their personal music files, for a period of 2 weeks. The authors found that 35% of the participants underestimated, and 38% overestimated their listening time. Listening levels were underestimated by 16% of the participants, and overestimated by 38%. Although no external dosimeter was required, participants received a loaner smartphone instead of using their own. This again potentially reduces the ecological validity of the study (i.e., the extent to which the results of a study can be generalized to real-life settings).

Research guided data collection through smartphone applications is becoming increasingly popular, and has been used for a variety of purposes in different age groups. For this study, we developed a smartphone application that was able to monitor listening to music through PLDs for a period of 35 days in a large population of adolescents. In contrast to other studies, participants did not need to carry an external dosimeter, and were able to use their own smartphone. As the application ran in the background for an extensive period of time, participants were not constantly reminded that data on listening habits were being collected, resulting in a high ecological validity. The first aim of present study was to evaluate music listening habits through PLDs in adolescents with a smartphone application. The second aim was to investigate the level of agreement between objectively measured, and self-reported listening habits.

## Methods

### Study design and population

This study was embedded in the Generation R Study, a population-based prospective cohort study from fetal life onwards in Rotterdam, the Netherlands. The design and population have been described previously [26]. Briefly, pregnant women with an expected delivery date between April 2002 and January 2006 were enrolled in the study (*n* = 9778) [26]. The children born from these pregnancies will be followed at least until young adulthood. Data collection in children and their parents includes questionnaires, interviews and routine visits to the research center in the Erasmus Medical Center. At the age of approximately 13 years, 7968 adolescents were invited for another examination phase, of whom 4949 visited the research center. All adolescents who visited the research center between mid May 2017 and the end of March 2019 were invited to participate in this smartphone sub study (*n* = 2929). To be eligible to participate, adolescents needed to have an Android smartphone, as the application was only designed for smartphones running the Android operation system. The results of the study will be presented in two parts. In the first part we will include all participants, whereas in the second part we will only include those participants who completed the postal questionnaire on music exposure through PLDs. The study was approved by the Medical Ethical Committee at Erasmus Medical Center. Written informed consent was obtained from all participants and both their parents. Participants received a small present for participation, there was no financial compensation.

### Smartphone application and exclusion criteria

A smartphone application for Android operating systems was developed to objectively measure listening habits, i.e. the frequency, listening time and the listening level. The application was overseen by the departments of Communication, Security, Legal Affairs and the Data Protection Officer of the Erasmus Medical Center. Several rounds of technical development and field testing were conducted before the application was implemented within the Generation R Study. The application was available through the Google Play store, and could be installed directly on participants’ smartphone. Minimal storage capacity was required, and the power consumption was low. Participants were able to use their own ear- or headphones. To log in, a unique username and password were required, provided during participants’ visit at the research center. After logging in, participants received a questionnaire in the application including questions about the brand and type of smartphone, the use of earphones or headphones for listening, and volume limit settings. When participants completed the questionnaire, the application started to collect data and ran constantly in the background for a period of 35 days. We expect listening habits to change during the day or week, but not over longer periods of time. Therefore, a monitoring period of 35 days was chosen. Every time music or a video was being played on the smartphone, a first timestamp was recorded at the start, and a second timestamp when the playback ended. The time between the two timestamps was used to calculate the duration of the listening session. During every playback, the application kept track of whether ear- or headphones were plugged in the audio jack of the smartphone (none of the participant used Bluetooth ear- or headphones). Only in case ear- or headphones were plugged in, the playback was considered as a listening session. Listening sessions lasting less than 1 min were excluded. In addition, some listening sessions showed a very long duration. It was unclear whether participants actually listened for such long time or it was a measurement error. Therefore, listening sessions lasting more than 8 h were excluded. This cut-off was based on available and relevant literature [20]. Besides data on the frequency of use and listening time, the application stored information on listening levels. Android smartphones generally have 15 listening levels, with 15 representing the maximum listening level. This scale was converted to a 0 to 100% scale, with 100% representing the maximum listening level. When participants changed the listening level to a lower or higher level during a listening session, it was saved by the application. Listening levels were averaged during the data analyses, taking the time of listening at a certain level into account. The application was not able to detect the volume limit settings. Volume limit lowers the maximum listening level but does not change the scale of the listening levels. Therefore, additional measures were not required during data analysis for participants who set a volume limit. Although the application period was intended to be 35 days, participants could delete the smartphone application at any time. Participants were included in the analyses if the time of installation, or time between first and last measurement, was at least 7 days with a maximum of 40 days. At the end of the monitoring period, when all data were collected, participants received an overview of their listening habits. No overview was provided when participants deleted the application before the 35 day period. The data recorded by the application was stored on the smartphone, and transferred to a secured online server every time the smartphone was connected to WiFi, to prevent data from being lost. The application did no store any other information than mentioned above.

### Questionnaire

Around the age of 13 years, participants were sent a postal questionnaire as part of the Generation R study. This extensive questionnaire contained three questions about music exposure through PLDs, among many other questions evaluating the health, growth and development of the participants. The first question was about the average number of days listening to music with ear- or headphone a week (never, 1–2 days, 3–4 days, 5 or more days a week). The second question asked about the average listening time on those days (< 30 min, 30 to 60 min, 1 to 2 h, 2 to 3 h, 3 to 4 h, and ≥ 4 h). The last question was about the usual listening level of the PLD (listening level at the lowest, at a quarter, half way, at three-quarters or at the highest level). Participants received the questionnaire before installation of the smartphone application but could send back the questionnaire at any time.

### Covariates

Demographic characteristics including participant’s sex, age, ethnicity, educational level, maternal educational level, and household income were obtained by parental questionnaire at different time points.

### Statistics

Statistical analyses were performed in IBM SPSS statistics version 24. Descriptive statistics were used to evaluate participants’ demographic characteristics and listening habits. Continuous data are described as mean (standard deviation (SD)) when normally distributed, or median (interquartile range (IQR)) when not normally distributed. Categorical variables are described as number (%). The continuous outcome measures of the smartphone application were categorized in the same way as the questionnaire responses. The kappa statistic was used to assess the level of agreement between the objectively and self-reported measured listening habits [27, 28] The weighted kappa was applied to take into account the extent to which there is disagreement. The kappa coefficient was interpreted using the guidelines outlined by Landis and Koch, where the strength of agreement is interpreted in the following manner: k < 0 poor; 0.00 ≤ k ≤ 0.20 slight; 0.21 ≤ k ≤ 0.40 fair; 0.41 ≤ k ≤ 0.60 moderate; 0.61 ≤ k ≤ 0.80 good; k > 0.8 very good [29].

## Results

**Fig. 1:**
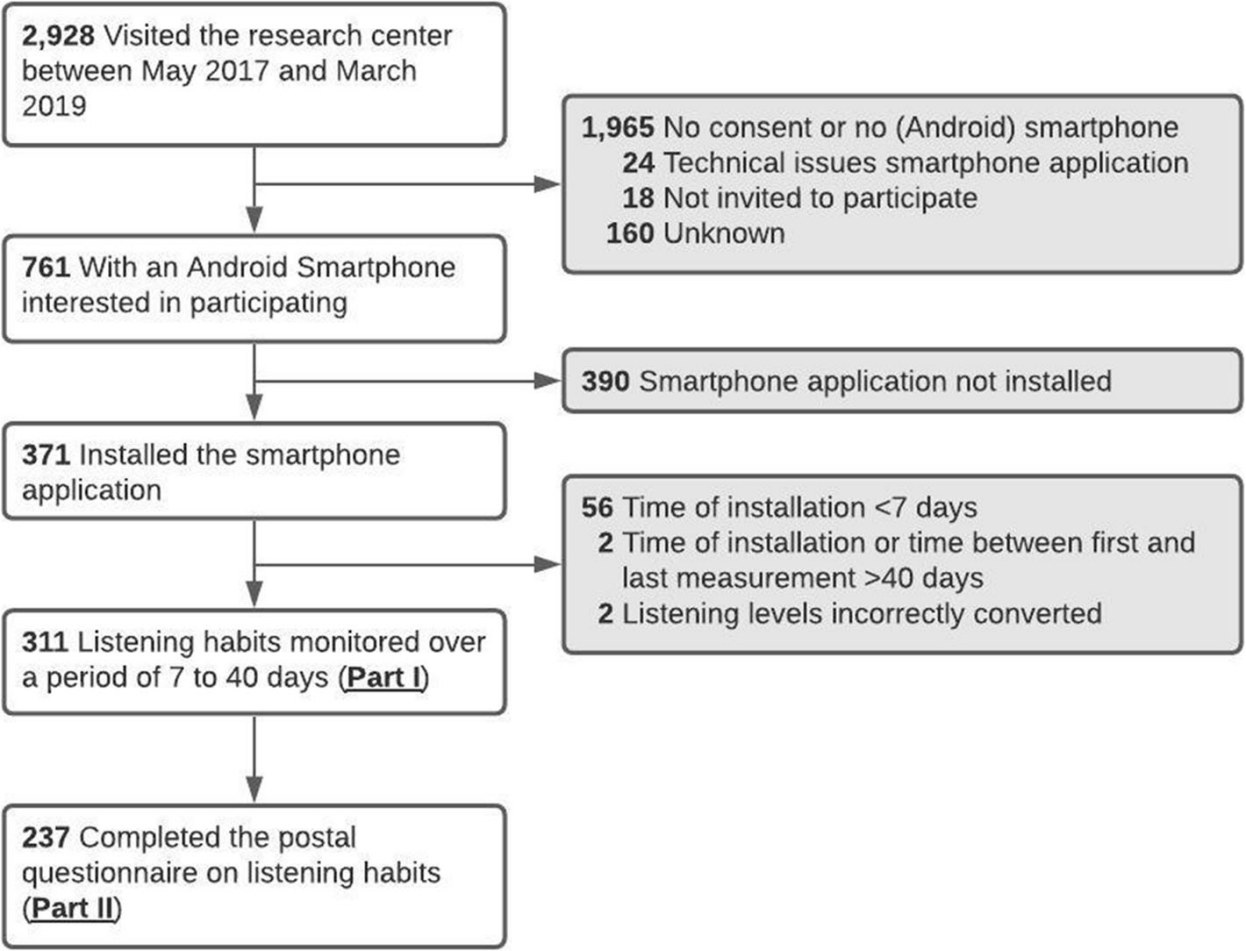
Flow chart of study sample

Of the 2928 adolescents visiting the research center between May 2016 and March 2019, 761 (26.0%) adolescents agreed to participate in this sub study. Main reasons for exclusion were no consent or not having a (Android) smartphone. Of the adolescents that agreed to participate, 371 (48.8%) actually installed, and started the application. Participants with an OnePlus smartphone were excluded as the listening levels were incorrectly converted (*n* = 2), i.e. listening levels above the maximum of 15. After excluding participants with an installation time or time between first and last measurement of less than 7 days or more than 40 days (*n* = 58), 311 adolescents were included in the first part of the study (Fig. 1). The postal questionnaire was completed by 237 out of 311 (76.2%) participants. These adolescents were included in the second part of the study. The mean age of all included participants was 13 years and 6 months (SD = 3 months, range = 12 to 15 years), and 161 (51.8%) were boys. The demographics of participants are demonstrated in Table 1. We investigated whether there was difference in demographic characteristics between the adolescents who were included in the analyses, and the adolescents who did not participate in this sub study (*n* = 4618). The participants included in the analyses were on average younger, more often Western, attended a higher level of education, and had higher educated mothers (Supplementary Table 1).

**Table 1:** Demographic characteristics of the adolescents included in the analyses

Characteristic	Smartphone application
	*n* = 311
Age, mean (SD)	13 y 6 mo (3 mo)
Gender, *n* (%)	
Male	161 (51.8)
Female	150 (48.2)
Ethnicity, *n* (%)	
Western	232 (74.6)
Non-Western	77 (24.8)
Unknown	2 (0.6)
Educational level, *n* (%)	
Low	69 (22.2)
Middle	49 (15.8)
High	161 (51.8)
Unknown	32 (10.3)
Maternal educational level, *n* (%)	
Low	11 (3.5)
Middle	118 (37.9)
High	169 (54.3)
Unknown	13 (4.2)
Household income, *n* (%)	
Low	64 (20.6)
Middle	108 (34.7)
High	91 (29.3)
Unknown	48 (15.4)

### Listening habits measured by the smartphone application

**Fig. 2:**
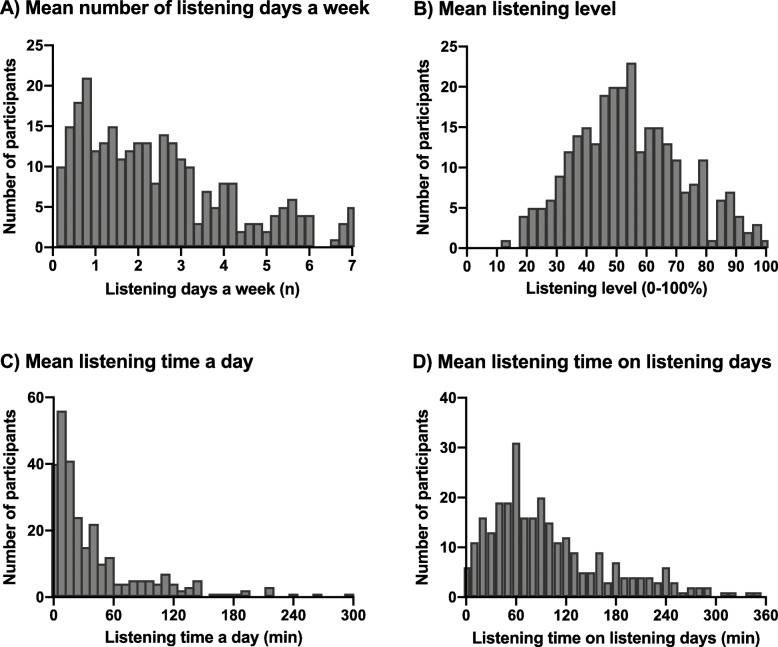
Distribution of mean number of listening days a week (**a**), mean listening time a day (**b**), mean listening time on listening days (**c**), and mean listening level (**d**) among the participants that had objective data on listening habits available (*n* = 282)

In the group of participants whose listening habits were monitored (*n* = 311), the most popular Android smartphone brands used were Samsung (65.9%), Huawei (13.5%) and Motorola (10.0%). The majority of participants used earphones (78.5%) instead of headphones (21.6%). Volume limit was used by 209 (67.2%) participants. In total, 282 (90.7%) participants had data on listening habits available, implying that they listened to music or watched a video with ear- or headphones on their smartphone at least once while the application was active. Listening habits were monitored over a course of a median of 33 days (IQR 23.8–35.0);2.8% of the participants had their listening habits monitored less than 10 days, 14.9% between 10 and 20 days, 17.4% between 20 and 30 days, and 64.9% between 30 and 40 days. The median number of days listening in a week was 2.1 (IQR 1.0–3.4). The median listening time was 21.1 min a day (IQR 9.1–53.7) when averaged over all days the application was installed, and 81.3 min (IQR 49.0–133.0) when only calculated over the listening days. The mean listening level was 54.5% (SD 18.1%). Figure 2 represents the distribution of the main outcome measures.

### Self-reported listening habits

Of the participants that had their listening habits monitored by the smartphone application, 237 (76.2%) completed the postal questionnaire. The majority of these participants (89.0%) reported using a PLD on a regular basis; 56 participants (23.6%) 1 to 2 days a week, 64 (27.0%) 3 to 4 days a week and 91 participants (38.4%) 5 or more days a week. On those days, the largest proportion of participants (34.6%) reported listening between 30 min and 1 h. Seven participants (3.0%) reported a listening time of more than 4 h on days listening to music. The greatest proportion of participants (47.7%) indicated that the listening level was on average 50% of the maximum listening level. Table 2 presents an overview of listening habits as measured by the smartphone application, and questionnaire.

**Table 2:** Listening habits as measured by the smartphone application (*n* = 311), and postal questionnaire (*n* = 237)

	Smartphone application	Postal questionnaire
Characteristic, n (%)	***n*** = 311	***n*** = 237
Listening days a week^a^		
Never	29 (9.3)	26 (11.0)
1–2 days a week	134 (43.1)	56 (23.6)
3–4 days a week	95 (30.5)	64 (27.0)
≥ 5 days a week	53 (17.0)	91 (38.4)
Listening time a day^b^		
< 30 min	193 (62.1)	–
30 min- 1 h	56 (18.0)	–
1–2 h	35 (11.3)	–
2–3 h	16 (5.1)	–
3–4 h	7 (2.3)	–
> 4 h	4 (1.3)	–
Listening time on listening days		
< 30 min	70 (22.5)	58 (24.5)
30 min- 1 h	58 (18.6)	82 (34.6)
1–2 h	102 (32.8)	60 (25.3)
2–3 h	37 (11.9)	22 (9.3)
3–4 h	28 (9.0)	8 (3.4)
> 4 h	16 (5.1)	7 (3.0)
Listening level		
< 25%	0 (0.0)	8 (3.4)
25%	53 (17.0)	29 (12.2)
50%	140 (45.0)	113 (47.7)
75%	75 (24.1)	42 (17.7)
100%	14 (4.5)	7 (3.0)
Unknown	29 (9.3)	38 (16.0)

^a^ No rounding was applied when calculating the number of listening days a week. If a participant exceeded the upper limit of a category, the participant was included in the next category. For example, when a participant listened on average 2.4 days a week it was considered as 3 days.

^b^ Outcome measure not included in the postal questionnaire

### Agreement between smartphone application and self-reported data

#### Listening days a week

Participants were asked about the average number of days they listened to music through their PLD with ear- or headphones. A slight agreement was found between the actual measured and self-reported number of days listening to music, k = 0.179 (95% CI, 0.095 to 0.262), *p* < 0.001 (Table 3). Forty-four participants (18.6%) underestimated the average number of days listening to music with ear- or headphones a week, whereas 109 (46.0%) overestimated their listening frequency. The questionnaire response of 84 participants (35.4%) was found to be consistent with their objectively measured data.

**Table 3:** Agreement between the results of the smartphone application and postal questionnaire

	N	Underestimation	Corresponded	Overestimation	kappa	95% CI	***p***-value
**Frequency**	237	44 (18.6%)	84 (35.4%)	109 (46.0%)	0.179	0.095 to 0.262	< 0.001
**Listening time**	237	105 (44.3%)	71 (30.0%)	61 (25.7%)	0.193	0.109 to 0.276	< 0.001
**Listening level**	185	36 (19.5%)	104 (56.2%)	45 (24.3%)	0.364	0.261 to 0.467	< 0.001

#### Listening time

Participants were questioned about the average listening time on those days listening to music with ear- or headphones. A slight agreement was found between the self-reported and objectively measured data, k = 0.193 (95% CI, 0.109 to 0.276), *p* < .001. A total of 105 (44.3%) participants underestimated the average listening time on listening days, whereas 61 (25.7%) overestimated their listening time. The questionnaire response of 71 participants (30.0%) corresponded to the objectively measured data.

#### Listening level

Participants were asked about the average listening level when listening to music with their PLD. There was fair agreement between the self-reported and objectively measured data, k = 0.364 (95% CI, 0.261 to 0.467), *p* < 0.001. Fifty-two (21.9%) participants did not respond to the question. Thirty-six participants (19.5%) of the 185 participants underestimated their average volume level, whereas 45 (24.3%) overestimated the listening level. The questionnaire response of 104 participants (56.2%) was found to be consistent with their objectively measured data.

#### Overall response questionnaire

When combining the results, we observed that 4 participants (1.7%) underestimated and 9 participants (3.8%) overestimated their listening habits at all three questions. In 16 (6.8%) participants the answers to all questions corresponded to the objectively measured data.

## Discussion

Listening to music through PLDs has become more prevalent during the last decades. Questions have been raised about the way PLDs are being used, and about possible damaging effects on hearing acuity. To date, the results of previous studies are mainly based upon self-reports, despite little evidence of its validity. In search of ways to objectively monitor music exposure, we developed a smartphone application that was able to collect data on listening habits in a large population-based cohort of adolescents over a substantial amount of time. In addition, the objectively measured exposure data serves a means to also validate self-reports.

Ninety percent of the adolescents in the present study used a PLD, which is in concordance with the literature [7–9, 30]. However, the listening habits measured by the smartphone application differed considerably from those of adolescents and young adults described by previous research using self-reports. We found that 17% of the participants listened 5 days or more a week, compared to 23.3 to 53.6% in other studies [7, 18, 20, 31]. In addition, 62.1% of the participants listened for less than half an hour per day, whereas previous studies reported an average or median listening time of more than 1 h a day [10, 21, 32]. In total, 3.6% of the participants listened for longer periods of time, more than 3 h a day, in comparison to 9.7 to 16.6% in other studies [7, 9]. The listening time on listening days and listening levels were generally similar to the results presented in literature [9, 10, 18, 20, 31–33]. The majority of the participants listened at a level between 50 and 75% of the maximum listening level. As the listening habits in the previously mentioned-studies were assessed by questionnaires, present results were also compared to the studies using objective measurement techniques. Kaplan-Neeman et al., using a loaner smartphone with a preinstalled application, reported that most 18 to 32 year old participants listen to music with their smartphone between 0.5 and 1.5 h on listening days, which is line with the results found in our study. Portnuff et al., using an external dosimeter to monitor listening habits, found that 19 to 29 year old participants listen on average 12.1 h per week, but with a wide range of 3.2 to 32.5 h. The listening time of the participants who listened for longer durations presumably skewed upward the cohort’s average. Furthermore, only participants who reported a minimum of 10 h of PLD use in a typical week were eligible to participate. Despite the fact that listening habits were measured objectively in both studies, the age range and measurement techniques differed from the one used in our study, which makes comparison difficult. For example, age is known to influence listening time and listening levels [7, 32]. Vogel et al. reported that adolescents aged 12 to 13 years take more breaks from music listening than adolescents aged 15 to 19 years, and are more likely to heed warnings against high listening levels [7]. Another explanation for the difference in results is that young people have adjusted their listening habits as a result of public awareness campaigns [17].

The level of agreement between the objectively measured and self-reported listening habits was assessed to investigate the accuracy of self-reports. Listening habits may vary over time, and change depending on listening conditions and surroundings [23, 31–33]. Questionnaires collecting data at a single time point might not account for this variability. In addition, they are prone to misreporting and bias. In this study, a slight to fair agreement between the objectively and self-reported listening habits was found. When participants were asked about the frequency and time of listening, the majority of participants overestimated the mean number of listening days a week (46.0%), and underestimated (44.3%) the mean listening time on listening days. This is probably just a reflection of random inaccuracy, rather than a meaningful trend. This seems not to be true for listening levels. These were reported most accurately, with consistency between the self-reported and objective measured data in 56.2% of the cases. Considering the low agreement between the objectively measured and self-reported data, these use of self-reports in the assessment of listening habits is questionable. We would advise to use a form of objective measurement of exposure data, such as the smartphone application used in this study.

There are a number of strengths and limitations. First, the current study was conducted as part of the Generation R Study, which has the advantage of having a large population based sample size. Second, a smartphone application was developed for our study in particular to collect real-time and dynamic data on listening habits in adolescents. The application was easy to use and required minimal storage capacity and battery. In contrast to other studies, our participants had the advantage that they did not need to carry an external or foreign device with them, which potentially reduces the ecological validity of the data [24, 25]. Furthermore, listening habits were monitored for a longer period compared to previous studies, which is of value since listening habits may vary over time. The primary limitation of this study is that only participants possessing a smartphone with Android operating systems were eligible to participate. This possibly induces selection bias, based on age, gender and socioeconomic status [34]. However, no difference in gender distribution and household income were found between the adolescents who were included in this sub study and adolescents who were not, but participants included in the analyses were on average younger, more often Western, attended a higher level of education, and had higher educated mothers. A second limitation is that the application could only be installed on a single PLD. If participants used multiple PLDs, it is likely that they overestimated their listening habits. Meanwhile, it is also possible that the smartphone was used by someone other than the participant, or that the ear-or headphones were not worn during a listening session. In that case, the application gives an over estimation of the actual use. Furthermore, it may be that participants used different types of ear- or headphones, or connected an external speaker to the output jack of the PLD, which affects the listening levels. Another limitation is that some listening sessions showed a long duration. As most of these listening sessions occurred during the day, we expect it to be a measurement error. It could be that the application was set to inactive or the smartphone was turned off during the listening session. Unfortunately, we were not able to track down the exact cause. We decided to exclude listening sessions lasting more than 8 h based on previous literature [20]. In future research, it would be of value to inquire participants about the maximum duration of a single listening session. In this study, we assume that the application has been running in the background without interruption. However, when the application was disabled by the Android operation system or participant, we did not receive a notification. To improve the certainty of the data, this feature will be included in future versions of the application.

With regard to the postal questionnaire, the are some limitations that needs to be discussed. As the postal questionnaire was not specifically designed for this sub study, there were certain discrepancies between the questions of the postal questionnaire and data collected by the smartphone application. Most important, the postal questionnaire asked about music exposure using any device (iPod, MP3 player or smartphone), whereas the application was only able to monitor music exposure through the smartphone. Yet, previous research has shown that smartphones are the most commonly used PLDs [10]. In addition, the smartphone application did not only measure music exposure but also other sources of noise exposure, such as watching videos and listening to audiobooks or podcasts. Another limitation is that the postal questionnaire and smartphone application did not necessarily evaluate the same time period. Participants were asked to complete the postal questionnaire just before installing the application. However, there were some participants who sent back the questionnaire after the data collection by the smartphone application was completed. This could have introduced bias, as participants received an overview of their listening habits at the end of the monitoring period of 35 days. Last, the participation rate was lower than expected, which potentially leads to selection bias. Yet, the response rate was sufficient to perform statistical analyses. The majority of adolescents did not participate as they did not give consent or did not have a (Android smartphone). A clarification of the lower response rate due to no consent could be the limited time at the research center allocated to inform and explain about the sub study with the smartphone application. We observed that the willingness to participate increased when more information was provided. Reminding the adolescents that wanted to participate but never installed the application after visiting the research center, resulted only in a few additional inclusions.

The smartphone application is currently being developed further, and will be available for smartphones running the iOS operating system in the near future. Listening habits will be examined, and we aim to investigate the potential risk of hearing loss from PLDs by combining data from the smartphone application and audiometric results. By studying the dose-response relationship, the magnitude of the risk for various exposure levels and patterns can be examined. When considering the acceptable noise dose from PLDs, other daily noise exposures should also be taken into account. Besides using the application for research purposes, it might also be of value in promoting safe listening behaviour. Information on personal usage can guide individuals on safe listening habits.

## Conclusions

This study describes a novel and innovative method to collect objective data on PLD use. Listening habits were monitored in a large population-based cohort of adolescents using a smartphone application in an easy and accurate way. We observed that the majority of participants used their PLD less frequently, and of shorter duration compared to what has been described in literature. The median number of listening days in a week was 2 days. The median listening time a day was 21 min. The accuracy with which PLD users report on their listening habits was also assessed in this study. A slight to fair agreement between the objectively and self-reported listening habits was found. In 30 to 56% of the cases, the response of the questionnaire was found to be consistent with the objectively measured data. Therefore, a smartphone application might be more reliable when examining listening habits, and could be of added value in future research evaluating the association between PLD use and hearing loss.

## Supplementary Information

**Additional file 1: Table S1.** Differences in demographic characteristics between the included and excluded participants.

## Data Availability

The data that support the findings of this study are available from Generation R but restrictions apply to the availability of these data, which were used under license for the current study, and so are not publicly available. Data are however available from the authors upon reasonable request and with permission of Generation R.

## Abbreviations

PLD: Personal listening device
dB(A): A-weighted decibel
