# Correction to: A smartphone application to objectively monitor music listening habits in adolescents

**DOI:** 10.1186/s40463-021-00532-y

**Published:** 2021-08-12

**Authors:** Danique E. Paping, Jantien L. Vroegop, Simone P. C. Koenraads, Carlijn M. P. le Clercq, André Goedegebure, Robert J. Baatenburg de Jong, Marc P. van der Schroeff

**Affiliations:** 1 Department of Otorhinolaryngology, Head and Neck Surgery, Erasmus University Medical Center, Rotterdam, The Netherlands; 2 The Generation R Study Group, Erasmus University Medical Center, Rotterdam, The Netherlands

**Correction to: J Otolaryngol Head Neck Surg 50, 11 (2021)**

**https://doi.org/10.1186/s40463-020-00488-5**

Following publication of the original article [1], the authors identified an error (omitted text) in Fig. 1. The correct figure is given below and the original article has been corrected.

**Fig. 1:**
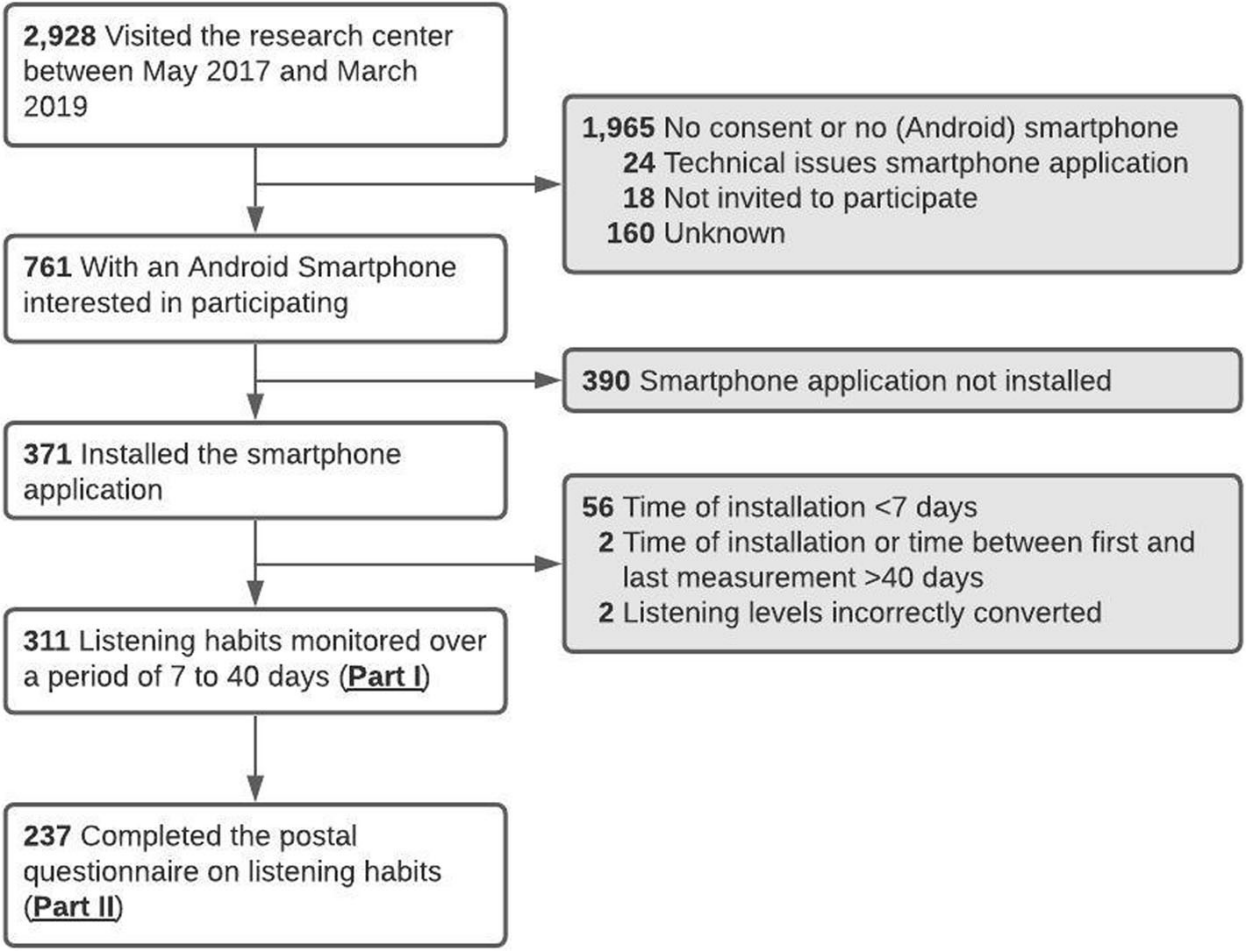
Flow chart of study sample
