# Supplementary material for: A smartphone application to objectively monitor music listening habits in adolescents: Personal listening device usage and the accuracy of self-reported listening habits
Source: J Otolaryngol Head Neck Surg. 2021 Feb 15;50:11. doi: 10.1186/s40463-020-00488-5 (PMC7885602; doi:10.1186/s40463-020-00488-5)
Supplement: Supplementary file 1 — Additional file 1: Table S1. Differences in demographic characteristics between the included and excluded participants. [file 40463_2020_488_MOESM1_ESM.docx]

**SUPPLEMENT**

**Table1^e^.** Differences in demographic characteristics between the included and excluded participants

 **Included Excluded p-value
Characteristic** n=311 n=4618
Age, mean (SD) 13 y 6 mo (3 mo) 13 y 8 mo (5 mo) 0.000
Gender, *n* (%)
 Male 161 (51.8) 2259 (48.9) 0.330
 Female 150 (48.2) 2359 (51.1)
Ethnicity^a^, *n* (%)
 Western 232 (74.6) 3179 (68.8) 0.034
 Non-Western 77 (24.8) 1333 (28.9)
 Unknown 2 (0.6) 106 (2.3)
Educational level^b^
 Low 69 (22.2) 979 (22.2) 0.003
 Middle 49 (15.8) 835 (18.1)
 High 161 (51.8) 2007 (43.5)
 Unknown 32 (10.3) 797 (17.3)
Maternal educational level^c^, *n* (%)
 Low 11 (3.5) 309 (6.7) 0.004
 Middle 118 (37.9) 1729 (37.4)
 High 169 (54.3) 2202 (47.7)
 Unknown 13 (4.2) 379 (8.2)
Household income^6^, *n* (%)
 Low 64 (20.6) 982 (21.3) 0.186
 Middle 108 (34.7) 1357 (29.4)
 High 91 (29.3) 1410 (30.5)
 Unknown 48 (15.4) 869 (18.8)

^a^ Western ethnicity included Dutch, European, American Western (including North American), Asian Western (including Indonesian and Japanese) and Oceanian. Non-Western ethnicity included Turkish, Moroccan, Surinamese, Antillean, Cape Verdean, African, Asian (except Indonesia and Japan) and South American and Central American.
^b^ Low educational level included primary education only or preparatory secondary vocational education (VMBO), middle included senior general secondary education (HAVO), and high included university preparatory education (VWO).  ^c^ Low educational level included no or primary education, intermediate included secondary school or vocational training, and high educational level included bachelor’s degree or university.
^d^ A net household income was classified as low (<2400 euros), middle (2400 to 4000 euros) or high (>4000 euros) based on total study population tertiles.

**Included Excluded p-value**

**Characteristic, *n* (%)**

**Completed postal questionnaire 237 (76.2) 3122 (67.6)**Listening days a week
 Never 26 (11.0) 435 (13.9) 0.092
 1-2 days a week 56 (23.6) 763 (24.4)
 3-4 days a week 64 (27.0) 638 (20.4)
 ≥5 days a week 91 (38.4) 1286 (41.2)
 Unknown 0
Duration on listening day
 < 30 minutes 58 (24.5) 993 (31.8) 0.003
 30 minutes- 1 hour 82 (34.6) 959 (30.7)
 1- 2 hours 60 (25.3) 631 (20.2)
 2- 3 hours 22 (9.3) 278 (8.9)
 3- 4 hours 8 (3.4) 132 (4.2)
 > 4 hours 7 (3.0) 117 (3.7)
 Unknown 0 12 (0.4)
Volume control setting
 <25% 8 (3.4) 91 (2.9) 0.997
 25% 29 (12.2) 376 (11.8)
 50% 113 (47.7) 1453 (46.5)
 75% 42 (17.7) 543 (11.3)
 100% 7 (3.0) 97 (3.1)
 Unknown 38 (16.0) 1064 (34.1)
